# Enhancement of Target-Oriented Opinion Words Extraction with Multiview-Trained Machine Reading Comprehension Model

**DOI:** 10.1155/2021/6645871

**Published:** 2021-03-27

**Authors:** Jingyuan Zhang, Zequn Zhang, Zhi Guo, Li Jin, Kang Liu, Qing Liu

**Affiliations:** ^1^Aerospace Information Research Institute, Chinese Academy of Sciences, Beijing 100190, China; ^2^Key Laboratory of Network Information System Technology (NIST), Aerospace Information Research Institute, Chinese Academy of Sciences, Beijing 100190, China; ^3^University of Chinese Academy of Sciences, Beijing 100049, China; ^4^School of Electronic Electrical and Communication Engineering, University of Chinese Academy of Sciences, Beijing 100190, China

## Abstract

Target-oriented opinion words extraction (TOWE) seeks to identify opinion expressions oriented to a specific target, and it is a crucial step toward fine-grained opinion mining. Recent neural networks have achieved significant success in this task by building target-aware representations. However, there are still two limitations of these methods that hinder the progress of TOWE. Mainstream approaches typically utilize position indicators to mark the given target, which is a naive strategy and lacks task-specific semantic meaning. Meanwhile, the annotated target-opinion pairs contain rich latent structural knowledge from multiple perspectives, but existing methods only exploit the TOWE view. To tackle these issues, we formulate the TOWE task as a question answering (QA) problem and leverage a machine reading comprehension (MRC) model trained with a multiview paradigm to extract targeted opinions. Specifically, we introduce a template-based pseudo-question generation method and utilize deep attention interaction to build target-aware context representations and extract related opinion words. To take advantage of latent structural correlations, we further cast the opinion-target structure into three distinct yet correlated views and leverage meta-learning to aggregate common knowledge among them to enhance the TOWE task. We evaluate the proposed model on four benchmark datasets, and our method achieves new state-of-the-art results. Extensional experiments have shown that the pipeline method with our approach could surpass existing opinion pair extraction models, including joint methods that are usually believed to work better.

## 1. Introduction

Target-oriented opinion words extraction (TOWE) [1] is a recently proposed subtask for fine-grained opinion extraction. In this task, entities or features mentioned in product reviews are treated as aspect targets, and text spans containing opinion expressions are regarded as opinion words. Given a target and the associated context, TOWE aims to extract opinion words that are related to a specified target. Examples of targets, opinions, and their relationship are shown in Table 1.

The core of the TOWE task is to infuse target-related knowledge into the model. TOWE is beyond identifying the boundary of all opinion mentions in product reviews. Instead, it further requires the ability to capture the association between opinions and the given target. Existing approaches mainly focus on learning target-aware contextualized representations to meet these requirements. For instance, Tang et al. [2] utilize the average embedding of target words to represent the target and leverage the concatenation of word and target embeddings as input to make the model aware of the given target. Directional information is also implicitly utilized by IOG [1], where tokens in the different sides of the target are first encoded separately and then aggregated with a recurrent network. Wu et al. [3] and Veyseh et al. [4] project the relative distance to the given target into embeddings as part of the model inputs. By informing the model with target knowledge, these methods achieve better performances than directly extract all opinions. However, these embedding-based or directional-based approaches to infuse target knowledge are weak and lack semantic information about the task. Thus, they still do not yield satisfactory results.

Another limitation that hinders the performances of existing works lies in that the latent structural knowledge in the opinion pair is underexploited. In addition to the perspective of building target-centric representations, learning correlation among context also contributes to TOWE. For example, by learning the conjunct relation among two opinions, these two phrases could benefit from each other when identifying the associated targets. State-of-the-art models, Zhang et al. [5] and Veyseh et al. [4], utilize the graph neural network to learn syntactical knowledge and capture opinion correlations. However, they rely on the dependency parser to generate graph structure, leading to a complex pipeline to train and deploy. This type of latent structure could also be modeled through transfer knowledge from the sentiment analysis task. Wu et al. [3] and Ying et al. [6] propose to capture latent opinion word distribution with sentence-level or aspect-level sentiment classification task and enhance the TOWE task with a transfer module or a joint training strategy. One drawback of this branch is their requirements for manually annotated sentiment classification resources, and they are unfeasible to be applied in new domains.

In this work, we present our MRC model trained from multiple perspectives to handle these issues. The main idea of QA is to learn a deep fusion of question and context to extract answer spans, which is in accord with the requirements of TOWE. Inspired by this observation, we formulate TOWE as a QA problem and leverage an MRC model to extract related opinion spans of a given target. Specifically, we leverage a template-based method to generate task descriptions as the pseudo-question and leverage a pretrained language model BERT [7] to learn deep interaction among this question and context to build target-aware representations. In this manner, we could obtain different context representations for each distinct target, and those expected opinion words could be identified based on these target-specific features. Another benefit is that the QA framework can be easily extended to more tasks (views) by changing templates with different task descriptions, which meets the requirements of our multiview learning paradigm.

Furthermore, we observe that the annotated target-opinion pairs could be used in multiple pathways to train the model. For example, these pairs could be extracted by identifying targets related to the given opinion phrase or classifying the relationship among a candidate target-opinion pair. We name these approaches to extract pairs of targets and opinions as different views of the same goal. Since these views could tackle the same task and share similar knowledge, it is natural to believe that training with correlated views could facilitate relation learning of the primary task. For instance, by extracting targets related to a given opinion, the model is implicitly aware of the latent semantic correlation among those targets, which is hard to capture for the TOWE view. Guided by this observation, we introduce a multiview training (MVT) framework to capture cross-view knowledge from multiple training perspectives. Specifically, we train the MRC model from three views: identifying opinions oriented to a given target, extracting opinion-related aspect targets, and pairwise relation classification. Since there are still discrepancies between selected views, directly apply multitask learning may introduce undesired noises. To alleviate this issue, we regard each view as a distinct learning task and utilize model-agnostic meta-learning (MAML) to capture common knowledge among tasks. The proposed MVT framework consists of two stages: first, aggregate knowledge from selected training views; second, we drive the final model through fine-tuning with TOWE data.

We evaluate the MRC-MVT framework on four widely used datasets for target-oriented opinion words extraction. Results from extensive experiments show that the introduced model outperforms existing works by a substantial margin, and we do not require extra annotated resources during training and inference stages. The main contributions of this work are summarized as follows:

1. We formulate TOWE as a QA problem and present a MRC model to handle this task. A template-based method is utilized to generate target-related task descriptions, and we exploit the machine reading comprehension ability of pretrained language to build high-quality target-aware representations.
2. To make the model better aware of the latent correlation among targets and opinions, we present a two-stage multiview training framework that first learns common knowledge from multiple views and then captures task-specific information for TOWE.
3. Experimental results demonstrate the effectiveness of the proposed MRC-MVT framework, and we achieve new state-of-the-art performances. Our method also performs well for target-opinion pair extraction when integrated with an aspect target detection module.

## 2. Related Work

### 2.1. Opinion Words Extraction

Extracting opinion expressions in reviews is a crucial step for fine-grained opinion analysis systems. Early works for opinion extraction typically leverage template-based methods such as associate rules [8] and syntactic rules [9] to match opinion words with predefined patterns. While these approaches are intuitive, manually designed or mined rules are sometimes too strict and lack generalization. Supervised learning methods typically treat opinion word extraction task as a sequence labeling problem. Lexical and syntactic features are utilized as inputs for conditional random fields (CRFs) [10, 11] and mine the sequential dependency of tokens. This task also benefits from the representation learning ability of neural networks and achieves good performances [12, 13]. It is also flexible for deep learning methods to learn the correlation between opinions and targets [14, 15], infuse syntactic knowledge [16], or even benefit from weakly annotated data [17].

In practice, opinion analysis seeks to extract pairs of targets and opinions. Several approaches first extract all opinion words and then learn the relation between targets and candidate opinions with rules [8, 18]. However, these methods do not yield satisfactory results. Recently, a more granular task, target-oriented opinion words extraction (TOWE), is introduced to fill in the gap. Instead of merely extracting all opinion expressions, TOWE is a collaborative process that identifies opinion words related to the given target. End-to-end neural methods [1] that directly take target information as inputs outperform those rule-based methods with a large margin. State-of-the-art methods [3–5] typically leverage extra information such as document-level sentiment representation and syntactic knowledge to assist the learning process. Nevertheless, existing works utilize position embedding to mark the target, which does not contain semantic information.

The idea of mining correlation among targets and opinions has also been explored in prior works. In [9], they propose a bootstrap method called double propagation (DP), which leverages manually defined rule and a small set of opinion words to extract opinions and targets iteratively. To get rid of dependency parser that could be inaccurate in reviews, TF-RBM [19] instead leverages sequential patterns to match targets of opinion phrases and introduces a two-fold method to obtain high-quality targets. However, there are two main differences between these methods and our approach: first, the proposed method could extract open-set opinion phrase while these two works either identify single word opinion or could only cover a fixed opinion dictionary; secondly, these two works are designed for the corpus-level task, while our method could extract desired information in a single sentence.

### 2.2. MRC-Based Information Extraction

MRC [20] is a fine-grained QA task that aims to extract answer spans in the context based on the given question. Numerous approaches [7, 21, 22] are proposed in recent years. The main idea of MRC is to understand the question and learn the correlation between context and question, thus identifying correct answers. MRC is a flexible paradigm to infuse information between the given question and context and is naturally adapted to information extraction tasks [23]. Instead of only extract one span per passage, these methods modify the prediction layer and leverage sequence labeling technique to hand potential multiple answers and achieve state-of-the-art results on named entity recognition [24], relation extraction [25], and event argument extraction [7, 26].

These works mainly investigate methods to build informative questions that contribute to identifying expected answer spans. For example, as suggested in [25], diverse questions templates yield better performances than a single question. In this work, we utilize the MRC-based model to extract target-oriented opinion words. Due to the feasibility of build questions, the proposed method could infuse target-related information and provide task descriptions to accelerate opinion extraction. This MRC paradigm also enables the multiview training procedure to handle multiple tasks in a unified framework by simply changing task descriptions and prediction heads. To the best of our knowledge, this is the first attempt that tackles fine-grained opinion extraction as a QA problem and leverages the MRC model to extract opinions related to a given target. Please note that the proposed method is not merely applying the MRC-based extraction framework. We further explore methods to train the MRC-based model from multiple views to enhance the primary task.

### 2.3. Multiview Learning

Multitask learning has been proven to be effective to learn share knowledge across tasks to improve performance of natural language tasks such as sentiment analysis [27–29], spoken language understanding [30], and named entity recognition [31]. Unlike the above methods that leverage extra resources as an auxiliary task, multiview learning is a branch of methods that focus on modeling the same task and dataset from different perspectives. The concept of multiview training frequently refers to learning and integrating knowledge from different input views. It has shown the ability to boost performance for multiple clustering [32] and classification [33, 34] tasks. The core of multiview learning is to build different views that reflect similar yet distinct types of information.

Besides those learn with multiple input views, MTMVL [35] also investigate methods to training a model from multiple views. This method formulates the primary task of relation extraction as multiple tasks and treats each task as a training view. By jointly training multiple views, the model could capture more relational knowledge from distinct perspectives and yield better performances than single view training. Our method is similar to this work, but there are three main differences: first, we tackle different problems with MTMVL; second, the proposed auxiliary views come from different but correlated tasks, while MTMVL leverages views for the same task; third, we further introduce a two-stage training framework and leverage meta-learning to learn common knowledge.

MAML [36] seeks to learn a model initialization trained on multiple tasks and could be adapted to the target task with a few annotated data points. It has been widely used in the field of computer vision, involving visual tracking [37], incremental object detection [38], and semantic segmentation [39]. MAML has also been applied to many NLP tasks such as text classification [40], named entity recognition [41], and relation extraction [42]. Recently, MAML has been utilized to reduce labeling noise for the semisupervised learning [43]. Different from existing works that mainly focus on few-shot learning, we explore the potential to capture common knowledge during the multiview training stage for target-oriented opinion words extraction.

## 3. Methodology

In this section, we present the framework of our MRC-MVT. We first show three ways to decompose the task of opinion pair extraction and the task formulation of these views in the Section 3.1 After that, we introduce our MRC model, which could solve these views in a unified framework. We show our two-fold multiview training strategy in the last subsection.

### 3.1. Task Definition

Three training views we utilized are listed in the following subsections. Note that auxiliary views are driven from the pairwise annotation structure and do not require extra human resources.

#### 3.1.1. Target-Oriented Opinion Words Extraction (TOWE)

TOWE is the primary view of this work to extract opinion words oriented to a specific target. Formally, given a sentence *X*={*x*_1_, *x*_2_,…, *x*_*n*_} with *n* tokens and an aspect target *T*={*x*_*j*_, *x*_*j*+1_,…, *x*_*k*_} where 1 ≤ *j* ≤ *k* ≤ *n*, we seek to tag each token *x*_*i*_ with a label *y*_*i*_, where *y*_*i*_ ∈ *𝒴*={*B*, *I*, *O*}. The first token of each corresponding opinion phrase is labeled with *B*, and other parts of opinion words are tagged with *I*; all nonopinion or irrelevant opinion words are annotated with *O*. Our model is required to produce tag sequence *Y*={*y*_1_, *y*_2_,…, *y*_*n*_} for *X* and extract correct opinion words. This view is a conditional extraction task where, for different targets of the same review, the corresponding tag sequence could also be different. TOWE requires the ability to identify opinions and estimate their relationships to the given target jointly. Examples of TOWE tagging results are shown in Table 2.

#### 3.1.2. Opinion-Related Aspect Targets Extraction (OATE)

Similar to TOWE, OATE is also a conditional extraction task. Given an opinion phrase *O*={*x*_*j*_, *x*_*j*+1_,…, *x*_*k*_}, where 1 ≤ *j* ≤ *k* ≤ *n*, we also utilize the sequence labeling technique to extract correlated aspect targets. OATE is the dual task of TOWE since it uses opinion phrases as inputs and seeks to extract aspect targets. The intuition behind this view is that if a model could extract correlated text spans for both directions, it could better capture relational knowledge among opinions and targets. Besides, this view could also contribute to learning implicit relations among targets, as shown in Table 1, where multiple targets could share the same opinion word. Labeling examples of OATE tagging results are shown in Table 3.

#### 3.1.3. Target-Opinion Pair Relation Classification (PRC)

Different from the other views, PRC is a classification task. Given an aspect target *T*={*w*_*j*_, *w*_*j*+1_,…, *w*_*k*_} and an opinion phrase *O*={*x*_*l*_, *x*_*l*+1_,…, *x*_*m*_}, this task seeks to classify if *O* is oriented to *T* in context *X*={*x*_1_, *x*_2_,…, *x*_*n*_}. Since both the target and the candidate opinion are given, this view does not need to extract candidate spans. It focuses on modeling the relationship between the given opinion and target explicitly. Examples of PRC classification results are shown in Table 4.

### 3.2. Machine Reading Comprehension Model

**Figure 1:**
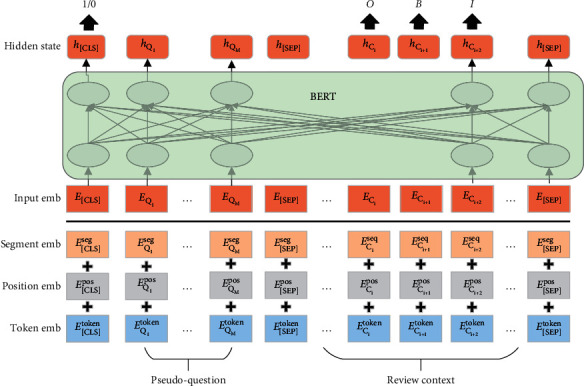
Architecture of the proposed MRC model. For the TOWE and OATE views, we tag each context token with a label. As for the PRC view, we classify the question-context pair based on the last BERT hidden state corresponding to the “[CLS]” token.

BERT [7] with multilayer transformer blocks is utilized as the backbone to learn contextualized representations for each token. The architecture of the MRC-based model is shown in Figure 1.

#### 3.2.1. Question Generation

We utilize a template-based strategy to generate view-specific questions for each view. Details are shown in Table 5. Each template composes of two parts: fixed view-specific text that describes task information and sentence-specific slots to be filled. Take an example from Table 2, to identify opinion words oriented to the target “service,” we fill the argument in template 1 with corresponding value and obtain the question “Opinion words oriented to aspect target service?.” These generated pseudo-questions are then utilized to inform the MRC model with task-relevant knowledge. We obtain task-specific and view-specific inputs of our MRC model with the following procedures:

1. The input sequence is padded with functional tokens “[CLS]” and “[SEP],” and the final input is formulated as “[CLS] [Question] [SEP] [Context] [SEP],” where “[Question ]” and “[Context]” are the constructed pseudo question and the original context, respectively.
2. The concatenated text is tokenized with WordPiece to handle the out-of-vocabulary problem. Those obtained subtokens are then mapped into word ids with a predefined lookup table.
3. Word ids, segment ids indicating whether the words are in the original text or not, and position ids indicating the absolute position starting from zero are utilized as inputs of our model. More details are shown in [7].

#### 3.2.2. Attention Interaction

Input representation of each token is constructed by summing word, segment, and position embeddings converted from input ids as shown in the bottom part of Figure 1. We initialize our model with BERT [7] to benefit from its large-scale pretraining corpus. Since the BERT encoder consists of multiple transformer blocks with the same architecture, we only briefly introduce one such block.

Input features corresponding to each token of the transformer block are first converted to query and key vectors of dimension *d*_*k*_, and value vector of dimension *d*_*v*_. A set of queries, keys, and values of the entail sequence is packed together to matrices *Q*, *K*, and *V*. Based on these inputs, the attention mechanism with scaled-dot production is utilized to learn contextualized knowledge. The output of the attention module is calculated as

(1) $$\begin{array}{c} \text{attention}\left(Q,K,V\right)=\text{softmax}\left(\frac{QK^{T}}{\sqrt{d_{k}}}\right)V. \end{array}$$

To enable the model to capture information from different representation subspaces, we apply multihead attention with *h* attention heads in parallel:

(2) $$\begin{array}{c} \text{MultiHead}\left(Q,K,V\right)=\text{Concat}\left({\text{head}}_{1},\ldots ,{\text{head}}_{h}\right)W^{O}, \end{array}$$

where head_*i*_=Attention(*QW*_*i*_^*Q*^, *KW*_*i*_^*K*^, *VW*_*i*_^*V*^) with parameter matrices *W*_*i*_^*Q*^, *W*_*i*_^*K*^ ∈ *ℝ*^*d*_model_×*d*_*k*_^, *W*_*i*_^*V*^ ∈ *ℝ*^*d*_model_×*d*_*v*_^, and *W*^*O*^ ∈ *ℝ*^*hd*_*v*_×*d*_model_^. The attended output for each token is then fed to a multilayer fully connected network to form the transformer block's final output. More details are shown in [44]. By stacking multiple such transformer blocks, the model is capable of learning complex interactions among question and context, thus building target-aware representations.

#### 3.2.3. Answer Prediction

We leverage the output hidden states *H* of the last transformer block as the final context representation. For sequence labeling views (TOWE and OATE), the probability distribution of token *i* is calculated as

(3) $$\begin{array}{c} P_{l}\left(Y_{i}|h_{i}\right)=\text{softmax}\left(W_{l}h_{i}+b_{l}\right), \end{array}$$

where *W*_*l*_ ∈ *ℝ*^*d*_model_×3^ and *b*_*l*_ ∈ *ℝ*^3^ are weight matrix and bias term, and we have 3 distinct tags. CrossEntropy [45] is utilized as our training criterion, and the optimizing objective of labeling task is formulated as

(4) $$\begin{array}{c} {ℒ}_{\text{Labeling}}=-\sum_{i=1}^{n}\sum_{j\in Y}I\left(y_{i}=j\right)\log\left(p\left(y_{i}|h_{i}\right)\right), \end{array}$$

where *𝕀*(*∗*)=1 if the formula in bracket is true, else *𝕀*(*∗*)=0.

For the PRC view, we utilize the last layer hidden state corresponding to “[CLS]” token as the sentence-level representation following [7]. The probability distribution of opinion pair relation is formulated as

(5) $$\begin{array}{c} P_{c}\left(Y_{i}|h_{i}\right)=\text{softmax}\left(W_{c}h_{\left[\text{CLS}\right]}+b_{c}\right), \end{array}$$

where *W*_*c*_ ∈ *ℝ*^*d*_model_×2^ and *b*_*c*_ ∈ *ℝ*^2^ are weight matrix and bias term, and there are 2 relation types. Similar to views mentioned above, CrossEntropy is also utilized in this view formulated as

(6) $$\begin{array}{c} {ℒ}_{\text{PRC}}=-\sum_{j\in \left\{0,1\right\}}I\left(y_{j}=j\right)\log\left(p_{c}\left(y_{j}h_{\text{cls}}|\right)\right), \end{array}$$

where *y*_*j*_ is the corresponding relation type of PRC task.

### 3.3. Multiview Training Framework

In this section, we describe our MVT paradigm. We first introduce MAML [36] in brief and then present our MAML-based training framework. The goal of MAML is to learn a parameter initialization that could be fast adapted to new tasks with a few training data. MAML typically consists of two subphases, meta-training, and meta-validation. For meta-training phase, given a base model *f*_*θ*_ with parameters *θ* and a learning task *𝒯*_*i*_ ~ *p*(*𝒯*), parameters of the adapted temporary model for *𝒯*_*i*_ are obtained through gradient update:

(7) $$\begin{array}{c} \theta_{i}^{\prime }=\theta -\alpha \nabla_{\theta }ℒi\left(f_{\theta }\right), \end{array}$$

where ℒ*i* is the loss function for *𝒯*_*i*_ and *α* is the learning rate for meta-training. The new parameter set *θ*_*i*_′ is then utilized for the meta-validation phase.

During meta-validation stage, to update parameters of the base learner, we optimize for the performance of *f*_*θ*_*i*_′_ with respect to *θ* across tasks sampled from *p*(*𝒯*). The meta-objective for validation phase is as follows:

(8) $$\begin{array}{c} \min_{\theta }\sum_{T_{i}∼p\left(T\right)}ℒi\left(f_{\theta_{i}^{\prime }}\right)=\sum_{T_{i}∼p\left(T\right)}ℒi\left(f_{\theta -\alpha \nabla_{\theta }ℒi\left(f_{\theta }\right)}\right). \end{array}$$

Please note that the meta-validation step is applied over the model parameters *θ*, whereas our base learner's objective is computed with the updated model parameters *θ*_*i*_′. The meta-optimization across tasks is performed via stochastic gradient descent (SGD), such that the model parameters *θ* are updated as follows:

(9) $$\begin{array}{c} \theta ⟵\theta -\beta \nabla_{\theta }\sum_{T_{i}∼p\left(T\right)}ℒi\left(f_{\theta_{i}^{\prime }}\right), \end{array}$$

where *β* is the step size of meta-validation. Details about the MAML are outlined in Algorithm 1.

**Figure 2:**
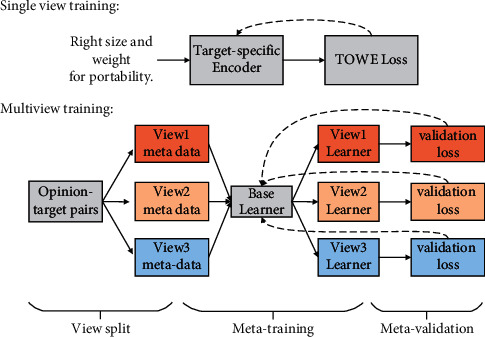
The overview of the proposed MVT framework is shown in this figure. Instead of directly optimize the model with TOWE task, we set the objective of base learner as minimizing the validation loss of adapted models to capture share knowledge among these views. Dash lines indicate the optimizing flows.

Similar to Zhang et al. [35], we jointly optimize the model with tasks from multiple views to capture shared knowledge, and the architecture of our method is shown in Figure 2. Though views we used are constructed from the same annotated corpus and share similar knowledge, there are still discrepancies between them. Directly performing multitask training may even introduce noise to the primary task. In this work, we leverage MAML to promote the MVT stage.

Different from the conventional meta-learning methods that seek for fast adaption to perform few-shot learning, our goal is to capture common knowledge across multiple views. We treat each of the proposed views as a distinct task and apply MAML over these tasks. We denote the MRC-based model as *f*_*θ*_. For each learning task, we sample a batch of support data *𝒟*_*i*_ and another disjoint batch of query data *𝒟*_*i*_′. Parameters *θ*_*i*_′ of the corresponding temporary model are obtained following equation (7), and we update the base model with equation (9).

The overall training process of our MRC-MVT framework is shown in Algorithm 2. We propose a two-step learning paradigm to fuse knowledge from multiple views. For the MVT stage, we explicitly encourage the base model to learn an initialization suitable for all of our training views, thus capturing shared information contained in each of these views. After that, we drive the final model by fine-tuning with the TOWE task based on parameters obtained with MVT to fit the goal of TOWE better.

## 4. Experiment

### 4.1. Datasets

We conduct experiments on 4 widely used benchmarks from SemEval Challenge 2014 task 4 (http://alt.qcri.org/semeval2014/task4/), SemEval Challenge 2015 task 12 (http://alt.qcri.org/semeval2015/task12/), and SemEval Challenge 2016 task 5 (http://alt.qcri.org/semeval2016/task5/). These datasets are original annotated with aspect targets and corresponding sentiment polarity. Opinion words that are oriented to the specific target are provided by [1], and we utilize the version with labeled with pairs of opinions for the following experiments. Statistics of these datasets are summarized in Table 6, and we randomly select 20% samples from the training set to build the development set.

### 4.2. Settings

Following [7], all sentences are tokenized with WordPiece. Our implementation is based on the Pytorch version of BERT (https://github.com/huggingface/transformers) with a 12-layer transformer, 12 attention heads, and 768-dimension hidden state. During training stage, we employ Adam [46] with *β*_1_=0.9 and *β*_2_=0.999 to finetune the model. The initial learning rate along with *β* and *γ* are set to 5*e* − 5, with a dropout rate of 0.1 and 100 warm-up steps. The maximum input word length is 128, and we set the training batch size to 32. We select *α* from {1*e* − 3,1*e* − 4} and MVT steps from {100,500,1000} based on the performances on development set. For fine-tuning stage, we optimize the model for 3 epochs.

### 4.3. Evaluation Metrics

We employ precision, recall, and *F*1 score as evaluating metrics following [1]. The performance is evaluated based on span-level matching. An extracted opinion word is considered to be correct only if the starting and ending positions are all exactly matched with the golden annotated ones. We report average results over three runs for the same setting based on the test set.

### 4.4. Main Results

We compare our method with the following works to evaluate our model:

1. Distance-rule [8] leverages the distance first rule to measure the relationship between opinions and targets. Intuitively, distance is a simple rule to measure the closeness between words. This method assigns the nearest token whose part-of-speech tag is adjective as opinion words.
2. Dependency-rule [18] takes part-of-speech tags, dependency path, and other linguistic features to mine rules on the annotated datasets. During the inference phase, these automatically mined templates are applied to match opinion words.
3. BiLSTM is an opinion word extraction approach: this method leverages the long-short-term memory network to extract contextualized representations for each token.
4. Pipeline first detects opinion words and then identifies their relationship with the given target. All opinion words are extracted with the BiLSTM network, and the distance-rule strategy is applied to assign the nearest opinion word to the given target.
5. TC-BiLSTM [2] is aware of target information to extract related opinions. The average embedding of the target phrase is concatenated with word embeddings as the final inputs to the model, and the BiLSTM model is used to learn representations.
6. IOG [1] employs two distinct BiLSTM networks to learn context representations from the different sides of the target. A global LSTM is then utilized to aggregate information from both sides and apply opinion word extraction.
7. LOTN [3] transfers document-level knowledge for the TOWE task. An attention-based model is first trained with a large-scale document-level sentiment classification dataset, and then latent opinion information is transferred to the TOWE model with knowledge distillation.
8. TS-GCN [5] is a syntax-based method that leverages GCN to capture dependency knowledge and utilizes a memory-based strategy to learn multiscale information.
9. ONG [4] is also a syntax-based method that incorporates the syntactic structures of the sentences through learning syntax-based opinion possibility scores and syntactic connections.

Results of baseline works and the proposed MRC-MVT are shown in Tables 7 and 8 . We utilize greedy decoding for all baselines and the proposed method to extract opinion phrases for a fair comparison following [1]. Results marked with ^†^ are reported by IOG [1].

From the first group of these two tables, we can observe that neural network methods consistently outperform rule-based strategies for all datasets. It is intuitively and reasonable because the expressions of opinion are complicated and the surface form varies. It is impractical to design rules that extract opinions with both high quality and recall. Due to their generalization ability, neural-based models achieve higher precision and recall.

Methods in the second group are neural models with target-specific information as inputs and directly extract opinions related to the given target. Benefiting from the joint extraction framework, these methods could avoid the error propagation problem between opinion extraction and relation identification process and thus achieve better performances. Notice that state-of-the-art methods LOTN, TS-GCN, and ONG utilize extra resources such as document-level sentiment classification dataset and syntactic structure. The proposed method still outperforms them with a large margin. Interestingly, the gains for recall are higher than those for precision. We attribute these gains to that our method is capable of learning implicit relations among opinion words by MVT.

In summary, the proposed method achieves *F*1 scores of 80.84, 87.83, 81.79, and 89.38 for 14lap, 14res, 15res, and 16res datasets, respectively. Our framework outperforms existing methods with an average gain of 4.23 points and achieves new state-of-the-art performance for the task of target-oriented opinion words extraction. These strong results demonstrate the effectiveness of our method.

### 4.5. Ablation Study

We conduct an ablation study to investigate the contribution of each component, and the results are shown in Table 9. The following variants are utilized to compare with the overall architecture:

1. ONG which achieves previously state-of-the-art performances on the utilized datasets.
2. MRC-MVT is our full method that applies MVT with a two-stage strategy.
3. Meta denotes the variant which only uses conventional multitask learning instead of leveraging meta-learning to fuse knowledge.
4. Post means we do not perform two-stage training and directly test the performance of our MRC model at the end of the MVT stage.
5. Views is the proposed based MRC model that does not apply MVT

The proposed MRC-based method achieves an absolute gain of 2.98 points over the previous state-of-the-art approach, leading to a strong baseline of our method. This result indicates that the MRC-based extracting model is a suitable choice for the TOWE task, and it is an efficient way to build target-centric representations and transfer knowledge from a pretrained language model. Compared with this strong baseline, training with our MRC-MVT strategy further leads to an average improvement of 1.25 points, which clearly shows the effectiveness of the proposed MVT framework.

Applying meta-learning during the MVT stage contributes to the model with 0.59 points. We attribute this gain to that meta-learning is a powerful way to aggregate common knowledge among these views and could reduce the effects of discrepancies among selected views. When removing the fine-tuning stage and directly test the MRC model at the end of the multi-vie training stage, the *F*1 scores dropped by 1.13 points. We can observe that although meta-learning contributes to learning a good initialization for the task of TOWE, fine-tuning on the final view is still required to fit our primary task better.

### 4.6. Performances with Different Views

**Figure 3:**
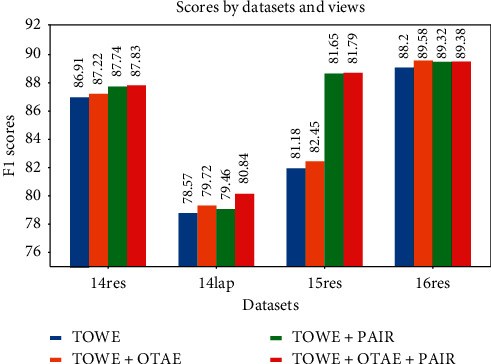
TOWE improvements with different views.

We also use different combinations of the proposed views to train the model, and results are shown in Figure 3. We can observe that all datasets could benefit from each of our proposed views. Adding the OATE view performs better than adding the PRC view and the combination of all three views achieves the best overall result. While the PRC task decides the relation based on the given target and opinion, OATE learns more knowledge to detect and classify aspect targets jointly. This may explain why the performances of adding the OATE view outperforms adding the PRC view. These two views also contain complementary knowledge, and the proposed method could benefit from jointly learning with both of them.

### 4.7. Performances on Target Extraction

We further conduct experiments on the target extraction task with the utilized dataset. We compare the performances of our method with the following baselines:

1. RNCRF [16] uses a tree-based recursive neural network to propagate syntactic information and decode aspect targets with conditional random fields.
2. CMLA [14] is a multilayer architecture where each layer consists of two coupled GRUs to model the relation between aspect terms and opinions.
3. HAST [47] is a sequential model that could selectively learn opinion information to enhance target representation.
4. SpanMlt-BERT [48]: a span-based joint approach that directly extracts target-opinion pairs based on pairwise span representations.

We utilize “aspect targets in this review” as the question for the target extraction task. As shown in Table 10, our method outperforms baseline methods and achieves new state-of-the-art results in target extraction. While our baseline model already obtains relatively high performance, the MVT framework's utilization further boosts the results, verifying the effectiveness of our method. As for SpanMlt-BERT, which also utilizes BERT as the base encoder, our method still surpasses it and obtains an average gain of 1.94 points. This result indicates that our method's improvement not only relies on the knowledge captured by BERT but also on our learning paradigm. Notice that this experiment further verifies the assumption that aggregating knowledge from various views and tasks could boost the performances of fine-grained opinion mining.

### 4.8. Performances on Pair Extraction

In real-world applications, the final outputs of an opinion extraction system should produce pairs of targets and opinions. In this subsection, we report the pair-wise *F*1 scores of the proposed method and existing systems as follows:

1. HAST + IOG is a pipeline approach where HAST [47] is first utilized to detect aspect targets and IOG [1] is then applied to extract related opinions.
2. JERE-MHS [49] is a joint entity and relation extraction model that could simultaneously extract aspect targets, opinion words, and their pairwise relations.
3. SpanMlt-BERT [48] is the previously state-of-the-art framework to jointly extract opinion-target pairs.
4. HAST-MRC utilizes HAST as the target detector and extracts related opinion words with the proposed method.
5. MRC-MVT utilizes the target extraction model, as shown in the previous subsection, to identify aspect targets and extracts related opinion words with the proposed MRC-MVT extractor.

As shown in Table 11, the proposed method consistently achieves the best pairwise results with large margins on the test set of all datasets. Compared with the best existing method, the proposed framework achieves an average gain of 2.59 points with HAST as the target extractor, and our full pipeline outperforms it with a margin of 4.98 points. From this observation, we can conclude that using our method could yield better overall performances for the pair extraction task. While recent works claim that joint learning strategies could achieve better performances to capture pairwise knowledge, our pipeline method beats those joint models. This result further demonstrates that our multiview learning method could effectively capture relational knowledge. Another observation is that the average performance gain in pair extraction is larger than that in pair extraction. We attribute this improvement to that our method could learn better relational knowledge than existing works. This indicates that identifying the correct targets is still a bottleneck of our pipeline, and existing joint models could be improved by enhancing the relational learning module.

## 5. Conclusions and Future Scopes

In this work, we introduce MRC-MVT, a new learning paradigm to extract opinion words oriented to a given target. We formulate this task as a QA problem and leverage a transformer-based pretrained model to produce target-aware representations for context words. Unlike existing methods that only focus on a single view, we further present a multiview training framework with meta-learning to aggregate common knowledge from different forms of the same task. The proposed method could benefit from the extended opinion extraction structure and make better use of existing datasets. Experiments on four widely used datasets validate the effectiveness of our MRC model, and we obtain new state-of-the-art results. When trained with the proposed MVT paradigm, the proposed method could achieve even better performances. These results clearly demonstrate the significance of our MRC-MVT framework. In the future, we would like to exploit our MVT framework's usage on opinion-related tasks such as aspect-based sentiment analysis. We also aim to build new end-to-end models that could directly extract pairs of opinions.

## Figures and Tables

**Algorithm 1:**
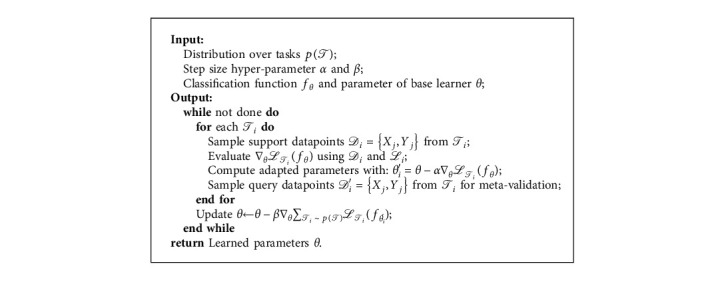
Algorithm for MAML.

**Algorithm 2:**
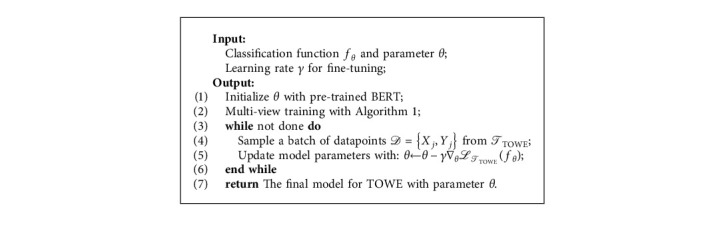
Algorithm for MRC-MVT framework.

**Table 1:** Examples for opinion-targets pairs.

**Review**: the operating system and user interface is very intuitive, and the large multitouch track pad is amazing.	
**Targets**	Operating system, user interface, multi-touch track pad
**Opinions**	Intuitive, amazing
**Pairs**	Operating system, intuitive
	User interface, intuitive
	Multitouch track pad, amazing

Targets are marked in Bold and corresponding opinions are in italics.

**Table 2:** Examples for TOWE view.

1. Go/*O* to/*O* Volare/*O* for/*O1*^*st*^/*Bclass*/*I ***service**/*O* and/*Oterrific*/*O ***food**/*O* ./*O*
2. Go/*O* to/*O* Volare/*O* for/*O* 1^st^/*O* class/*O* service/*O* and/*Oterrific*/*B ***food**/*O* ./*O*

Opinions are marked in bold and corresponding targets are in italics.

**Table 3:** Examples for OATE view.

1. Most/*O* everything/*O* is/*O* **fine**/*O* with/*O* this/*O* machine/*Ospeed*/*Bcapacity*/*Bbuild*/*B* ./*O*
2. It/*O* even/*O* has/*O* a/*O* **great**/*O* **webcam**/*B* ,/*O* and/*OSkype*/*O* works/*O* very/*O ***well**/*O* ./*O*
3. It/*O* even/*O* has/*O* a/*O* great/*O* webcam/*O* ,/*O* and/*OSkype*/*O* works/*O* very/*O ***well**/*O* ./*O*

Opinions are marked in bold and corresponding targets are in italics.

**Table 4:** Examples for PRC view.

Label	Context
1	The **bread** was *stale*, the salad was overpriced and empty
0	The **bread** was stale, the salad was *overpriced* and empty
0	The **bread** was stale, the salad was overpriced and *empty*
0	The bread was *stale*, the **salad** was overpriced and empty
1	The bread was stale, the **salad** was *overpriced* and empty
1	The bread was stale, the **salad** was overpriced and *empty*

**Table 5:** Templates to generate questions. “[TARGET]” and “[OPINION]” are argument placeholders to be filled with aspect target and opinion phrases, respectively.

Views	Question template
TOWE	1. Opinion words oriented to aspect target [TARGET]?
OATE	2. Aspect targets related to opinion word [OPINION]?
PRC	3. Is opinion word [OPINION] oriented to aspect target [TARGET]?

**Table 6:** Statistic of experiment datasets.

Dataset	Split	Sentences	Targets
14lap	Training	1158	1634
	Testing	343	482

14res	Training	1627	2643
	Testing	500	865

15res	Training	754	1076
	Testing	325	436

16res	Training	1079	1512
	Testing	329	457

**Table 7:** Comparison with existing works.

Models	14lap			14res		
	*P*	*R*	*F*1	*P*	*R*	*F*1
Distance-rule^†^	50.13	33.86	40.42	58.39	43.59	49.92
Dependency-rule^†^	45.09	31.57	37.14	64.57	52.72	58.04
LSTM^†^	55.71	57.53	56.52	52.64	65.47	58.34
Pipeline^†^	72.58	56.97	63.83	77.72	62.33	69.18
TC-BiLSTM^†^	62.45	60.14	61.21	67.65	67.67	67.61
IOG^†^	73.24	69.63	71.35	82.85	77.38	80.02
LOTN	77.08	67.62	72.02	84.00	80.52	82.21
TS-GCN	72.37	73.89	73.12	83.38	83.30	83.34
ONG	73.87	77.78	75.77	83.23	81.46	82.33
MRC-MVT	79.59	82.12	80.84	86.31	89.42	87.83

**Table 8:** Comparison with existing works (2).

Models	15res			16res		
	*P*	*R*	*F*1	*P*	*R*	*F*1
Distance-rule^†^	54.12	39.96	45.97	61.90	44.57	51.83
Dependency-rule^†^	65.49	48.88	55.98	76.03	56.19	64.62
BiLSTM^†^	60.46	63.65	62.00	68.68	70.51	69.57
Pipeline^†^	74.75	60.65	66.97	81.46	67.81	74.01
TC-BiLSTM^†^	66.06	60.16	62.94	73.46	72.88	73.10
IOG^†^	76.06	70.71	73.25	85.25	78.51	81.69
LOTN	76.61	70.29	73.29	86.57	80.89	83.62
TS-GCN	81.08	75.65	78.28	85.15	83.04	84.08
ONG	76.63	81.14	78.81	87.72	84.38	86.01
MRC-MVT	82.04	81.54	81.79	90.60	88.19	89.38

**Table 9:** Ablation study.

Variants	14lap	14res	15res	16res	Average
ONG	75.77	82.33	78.81	86.01	80.73
MRC-MVT	80.84	87.83	81.79	89.38	84.96

Meta	79.47	87.65	81.44	88.93	84.37
Post	79.05	86.54	80.93	88.78	83.83
Views	78.57	86.91	81.18	88.20	83.71

**Table 10:** Performance on target extraction.

Models	14lap	14res	15res	16res	Average
RNCRF	74.92	75.18	74.14	73.12	74.34
CMLA	75.57	76.08	78.31	76.84	76.70
HAST	79.14	82.56	79.84	81.44	80.74
SpanMlt-BERT	80.78	84.26	77.71	80.95	80.92
MRC	80.31	86.73	77.85	81.59	81.62
MRC-MVT	82.64	87.15	78.42	83.24	82.86

**Table 11:** Performance on pair extraction.

Models	14lap	14res	15res	16res	Average
HAST + IOG	53.41	62.39	58.12	63.84	59.44
JERE-MHS	52.34	66.02	59.64	58.58	59.14
SpanMlt-BERT	65.75	72.72	61.06	69.58	67.27
HAST + MRC	64.71	73.13	67.92	73.68	69.86
MRC-MVT	67.35	77.02	68.63	75.99	72.25

## Data Availability

The data used to support the findings of this study are available at https://github.com/NJUNLP/TOWE.
